# Cine CMR diastolic function parameters in acute ST-elevation MI (STEMI) patients are associated with cardiac injury and left ventricular strain

**DOI:** 10.1186/1532-429X-13-S1-P93

**Published:** 2011-02-02

**Authors:** Ana Barac, Gopal Ghimire, Jyotshana Shrestha, Manuel A Gonzalez, William O Suddath, Augusto D Pichard, Lowell F Satler, Ron Waksman, Anthon R Fuisz, Gaby Weissman

**Affiliations:** 1 Washington Hospital Center/Georgetown University Hospital, Washington, DC, USA; 2 Washington Hospital Center, Washington, DC, USA

## Objective

The aim of this study was to investigate associations between different parameters of diastolic function assessed from cine CMR, biomarkers of cardiac injury and left ventricular function and strain in patients with STEMI.

## Background

Presence of diastolic dysfunction in patients with history of myocardial infarction represents a marker of adverse outcomes. However, its role in acute ST-elevation myocardial infarction (STEMI) is not fully elucidated. Cardiovascular magnetic resonance (CMR) allows quantitative measurement of volumetric changes that occur during diastole and form basis for calculation of different parameters of diastolic function. The relationships between these parameters, LV systolic function and strain, and markers of cardiac injury in STEMI are unknown.

## Methods

Thirty patients with acute STEMI undergoing primary PCI were prospectively enrolled and underwent CMR imaging and 2D echocardiography within 48 hours of admission. CMR images were planimetered (Qmass, Medis, The Netherlands) in all slice positions across all temporal phases to calculate diastolic parameters: E/A ratio, peak filling rate (PFR and nPFR-normalized value for stroke volume), time to peak filling rate (TPFR), and diastolic volume recovery (DVR_80_ - proportion of diastole required to recover 80% stroke volume). Longitudinal, radial and circumferential strain were assessed from standard echocardiographic views using 2D speckle-tracking software (2D CPA, TomTec, Germany). All patients had serial troponin values (at least three) measured.

## Results

**Figure 1:**
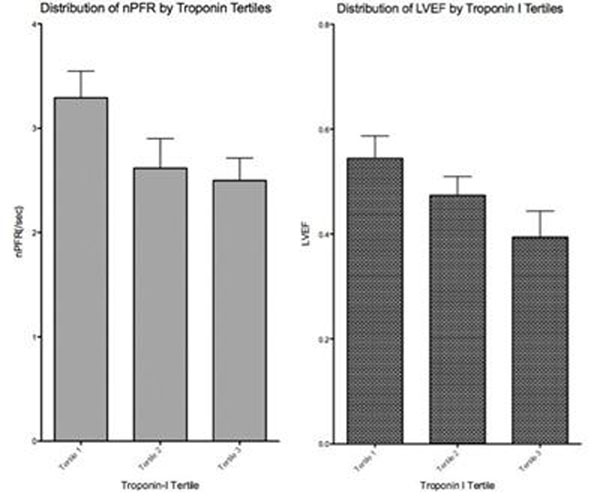


Median troponin value was 34ng/mL (IQR 15.8-101.8) and median time from symptom onset to reperfusion was 194 minutes (IQR 158-277). Normalized PFR and DVR_80_ were associated with peak troponin I levels (r=-0.36, P=0.049, and r=-0.41, P=0.011 respectively). LV ejection fraction as well as global LV longitudinal strain correlated with nPFR (r=0.432, P=0.017 and r=-0.565, P=0.010 respectively) but the association with DVR_80_ was not significant (r=0.277, P=0.18 and r=-0.232, P=0.24, respectively). Patients in the lowest time to reperfusion tertile had significantly higher nPFR compared to patients in the highest tertile (3.32+0.84 s^-1^ vs 2.27+0.52 s^-1^). Figure 1.

## Conclusions

In acute STEMI patients diastolic function parameters by cine CMR imaging predict the extent of cardiac injury and are associated with the time to reperfusion. The role of these new measures of myocardial function in prognosis post STEMI remains to be investigated.

